# Characterisation of Haemolytic Uropathogenic *Escherichia coli* Isolated From Dogs and Cats

**DOI:** 10.1002/vms3.71200

**Published:** 2026-08-31

**Authors:** Sarah Büttner, Joachim Spergser, Olga Makarova, Michael P. Szostak, Adriana Cabal Rosel, Werner Ruppitsch, Sabine Schäfer‐Somi, Elke Müller, Sascha D. Braun, Stefan Monecke, Ralf Ehricht, Frank Künzel, Igor Loncaric

**Affiliations:** ^1^ Institute of Microbiology University of Veterinary Medicine Vienna Austria; ^2^ Centre for Food Science and Veterinary Public Health University of Veterinary Medicine Vienna Austria; ^3^ Division for Public Health, Austrian Agency for Health and Food Safety Institute for Medical Microbiology & Hygiene Vienna Austria; ^4^ Institute of Hygiene and Medical Microbiology Medical University Innsbruck Innsbruck Austria; ^5^ Department of Small Animals and Horses, Clinical Center for Reproduction University of Veterinary Medicine Vienna Austria; ^6^ Leibniz‐Institute of Photonic Technology (Leibniz‐IPHT) Leibniz Center for Photonics in Infection Research (LPI) Jena Germany; ^7^ InfectoGnostics Research Campus Jena Germany; ^8^ Institute of Physical Chemistry Friedrich‐Schiller‐University Jena Germany; ^9^ Department for Companion Animals and Horses Clinic for Small Animals, University of Veterinary Medicine Vienna Vienna Austria

**Keywords:** cats, dogs, *E. coli*, ESBL, ExPEC, multi‐drug resistance, ST131, UTI, UPEC, virulence

## Abstract

**Background:**

*Escherichia* (*E*.) *coli* is the primary cause of urinary tract infections in companion animals.

**Objectives:**

Therefore, the present study aimed to characterise antimicrobial resistance profiles, virulence‐associated genes, and phylogenetic diversity of *E. coli* with haemolytic colony morphology isolated from urine samples of cats and dogs during routine diagnostic activities, which have not been characterised previously in Austria.

**Methods:**

A total of 51 isolates were included from dogs (*n* = 32) and cats (*n* = 19) and characterised by a polyphasic approach, including susceptibility testing and DNA microarray‐based assays. In addition, all isolates were analysed using two‐locus *fumC* and *fimH* sequence typing (CH‐clonotyping) and *E. coli* phylotyping.

**Results:**

The majority of *E. coli* isolates belonged to phylogenetic group B2, with one isolate assigned to phylogenetic group B1. Thirty‐four CH‐clonotypes were detected using two‐locus sequence typing. All isolates detected could be classified as ExPEC.

**Conclusions:**

The identification of clonal complexes common to humans indicates interspecies transmission, thereby emphasising the critical need for surveillance in companion animal populations.

## Graphical abstract

The present study characterises 51 canine and feline haemolytic *E. coli* isolates from urinary tract infections by a polyphasic approach, including susceptibility testing, CH‐clonotyping, and DNA microarrays. The majority of isolates belonged to phylogenetic group B2 and were classified as extraintestinal pathogenic *E. coli* (ExPEC), exhibiting high clonal diversity. The presence of clonal complexes shared with humans suggested potential interspecies transmission, highlighting the need for surveillance of companion animals as reservoirs for these pathogens

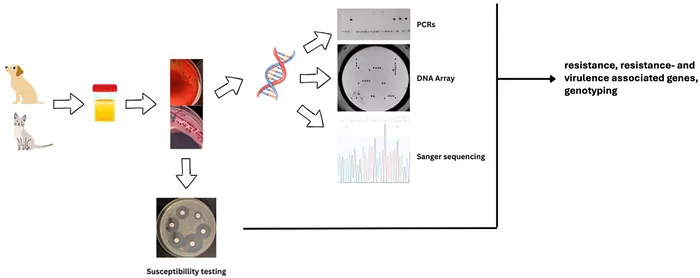

## 1 Introduction

*Escherichia* (*E*.) *coli* plays a significant role in acute infections in humans and pets, both gastrointestinal and extraintestinal (Tchesnokova et al. 2016). The pathotype of extraintestinal pathogenic *E. coli* (ExPEC) can be divided into different subpathotypes, such as uropathogenic *E. coli* (UPEC), avian pathogenic *E. coli* (APEC), and sepsis‐associated *E. coli* (SPEC), among others, based on phylogenetic group affiliations and a combination of virulence factors (Hutton et al. 2018). Infections caused by ExPEC include urinary tract infections (UTIs), sepsis, pneumonia, meningitis, skin and soft tissue infections, and osteomyelitis (Hutton et al. 2018). With infections of the urogenital tract being the reportedly most frequently diagnosed infectious disease in dogs, UPEC causes the majority of UTIs in both humans and animals (Kidsley et al. 2020; Aurich et al. 2023). The pathogenicity of UPEC arises from virulence mechanisms that enable it to colonise and proliferate in the epithelial cells of the bladder (Kidsley et al. 2020). This includes fimbrial adhesins, most commonly Type 1 fimbriae, which are extracellular protein structures that allow UPEC to attach to bladder cells (Whelan et al. 2023; Lüthje and Brauner 2014). Ristow and Welch (2016) describe alpha‐haemolysin as a commonly observed virulence factor in severe cases of UTIs in humans, with a correlation between the expression of haemolysin and the severity of infection. Alpha‐haemolysin (HlyA) (encoded by the *hlyCABD* operon) causes the lysis of red blood cells and exhibits cytotoxic activity against a variety of species and cell types. Extensive biochemical, electrophysiological, and structural studies using whole cells, erythrocytes, artificial membranes, and lipid bilayers have thoroughly demonstrated the pore‐forming action of HlyA in *E. coli*. Experimental data consistently show that HlyA inserts into target cell membranes, creating defined transmembrane pores. This action results in the lysis of the target cell due to the loss of its membrane integrity. Crucially, the detailed mechanism, including the dimensions, conductance properties, and precise process of pore formation, has been fully characterised through these experiments (Ristow and Welch 2016). Ristow and Welch indicate that approximately 50% of UPEC encode HlyA. Still, with increasing severity of infection, the proportion of the population expressing HlyA increases, with up to 78% of cases of pyelonephritis caused by UPEC encoding HlyA (Ristow and Welch 2016). In addition, *hlyA* genes in *E. coli* isolates were also associated with UTIs from companion animals (Ksiezarek et al. 2021; Siqueira et al. 2009; Siqueira et al. 2021; Melo et al. 2022; Jousserand et al. 2025; Valat et al. 2020). Therefore, the aim of the present study was to analyse *E. coli* with haemolytic activity on sheep blood agar isolated from urine samples of dogs and cats with signs of UTIs.

## 2 Materials and Methods

A total of 51 haemolytic *E. coli* isolates were collected between 2017 and 2023 during diagnostic activities at the Institute for Microbiology of the Veterinary University in Vienna. All these clinical samples were received from third parties and, therefore, are not subject to the reporting obligations of the Ethics and Animal Welfare Commission of the University of Veterinary Medicine in Vienna. Isolates were stored at −80°C in cryoprotectant medium for further characterisation. Isolates were obtained from urine samples of dogs (*n* = 32) and cats (*n* = 19) with infections of the urogenital tract, predominantly cystitis (*n* = 15), followed by haematuria (*n* = 10) and dysuria (*n* = 6) (Table 1 and Table S1). The isolates were recultivated on 5% sheep blood agar, and DNA was extracted using the GenEluteTM Mammalian Genomic DNA Miniprep Kits by Sigma‐Aldrich (Sigma‐Aldrich by Merck kGaA, Darmstadt, Germany), according to the manufacturer's instructions. Antimicrobial susceptibility testing was performed by agar disc diffusion according to the CLSI standards (Clinical and Laboratory Standards Institute 2018). *E. coli* ATCC 25922 served as the quality control. Discs containing the following antimicrobial agents were used: ampicillin (10 µg); cefotaxime (30 µg); ceftazidime (30 µg); cefoxitin (30 µg); meropenem (10 µg); gentamicin (10 µg); tobramycin (10 µg); amikacin (30 µg); ciprofloxacin (5 µg); trimethoprim–sulfamethoxazole (1.25/23.75 µg); tetracycline (30 µg); chloramphenicol (30 µg); fosfomycin (200 µg); and nitrofurantoin (300 µg) (Becton Dickinson; Heidelberg, Germany). Due to the absence of these genes in the DNA microarray panel, isolates showing resistance to tetracycline or chloramphenicol were further tested for the presence of the *tet*(A) and *tet*(B) or *cat* resistance genes using PCR (Grünzweil et al. 2021 May 31). The quinolone resistance‐determining regions (QRDR) of *gyrA* and *parC* in ciprofloxacin‐resistant isolates were amplified by PCR and sequenced (Everett et al. 1996). Isolates were phylotyped using the quadruplex assignment method as previously described (Clermont et al. 2013 Feb). Clonal groups of *E. coli* isolates were determined by two‐locus sequence typing by combining data from *fumC* and *fimH* sequences (“CH‐clonotyping”) (Weissman et al. 2012), using CHTyper (Roer et al. 2018) hosted at the Center for Genomic Epidemiology (https://cge.cbs.dtu.dk/services/chtyper/, accessed on 1.10.2025). In addition to clonotyping, in an isolate displaying unique characteristics (i.e., ESBL phenotype) and belonging to CH40‐30, seven‐locus multi‐locus sequence typing (MLST) (Wirth et al. 2006) and an allele‐specific PCR were performed to identify the O25b clone of *E. coli* (Clermont et al. 2009). A set of virulence‐associated genes (Table S2) was determined using a custom‐made DNA microarray‐based technology from INTER‐ARRAY (INTER‐ARRAY by fzmb GmbH, Bad Langensalza, Germany) according to the manufacturer's instructions (Monecke et al. 2007, Bernreiter‐Hofer et al. 2021). Isolates resistant to ampicillin or displaying an ESBL phenotype were further analysed by the INTER‐ARRAY Genotyping Kit CarbaResist (INTER‐ARRAY by fzmb GmbH; Bad Langensalza, Germany; all information upon request under https://www.inter‐array.com/products/). All resistance‐associated genes integrated into the INTER‐ARRAY Genotyping Kit CarbaResist array were as previously described (formerly CarbDetect AS‐2) (Braun et al. 2018).

**TABLE 1:** Summarised molecular characterisation, antimicrobial resistance, and virulence profile of the *E. coli* isolates investigated.

						Resistance profile	
Isolates	Host	Diagnosis	Phylo‐group	CH‐clonotyping	Virulence genes	Phenotype	Genotype
1430, 1431	Cat	Dysuria	B2	24‐102	*cnf1, pic, fimH, papC, hlyA, iucD*	NR	
1355	Cat	Cystitis	B2	14‐1463	*cnf1, fimH, hlyA*	AMP, STX	*bla*_TEM_, *bla*_OXA‐2_, *sul2*, *dfrA5*
1619	Cat	Chronic kidney disease	B2	14‐2	*cnf1, fimH, hlyA*	NR	
1319, 1594	Dog	Haematuria (1319), cystitis (1594)	B2	43‐1561	*cnf1, fimH, hlyA*	NR	
2164	Cat	Haematuria	B2	52‐286	*cnf1, fimH, papC, hlyA, iucD*	NR	
2428	Dog	Cystitis	B2	24‐1431	*cnf1, pic, fimH, hlyA*	NR	
2455, 4160, 3157	Dog	Pyelonephritis (2455), cystitis (4160), haematuria (3157)	B2	103‐9	*cnf1, fimH, hlyA*	NR	
2712	Dog	Not known	B2	103‐1629	*cnf1, fimH, hlyA*	AMP	*bla*_TEM_
2790	Cat	Not known	B2	13‐223	*cnf1, fimH, hlyA*	NR	
2771	Cat	FLUTD	B2	13‐5	*astA, cnf1, fimH, hlyA*	NR	
2721, 3616	Dog	Not known (2721), cystitis (3616)	B2	103‐9	*astA, cnf1, fimH, hlyA*	NR	
1177, 3617	Dog	Cystitis (1177), not known (3617)	B2	13‐9	*astA, cnf1, fimH, hlyA*	NR	
970	Cat	Cystitis	B2	13‐9	*cnf1, fimH, hlyA*	NR	
959	Dog	Haematuria	B2	24‐306	*cnf1, pic, fimH, papC, hlyA, iucD*	NR	
747	Cat	Dysuria	B2	14‐10	*cnf1, pic, fimH, papC, hlyA, iucD*	NR	
350	Dog	Cystitis	B2	24‐2	*cnf1, fimH, hlyA*	NR	
189	Cat	Pyelonephritis	B2	14‐9	*cnf1, pic, fimH, hlyA*	NR	
325	Dog	Not known	B2	13‐2	*cnf1, fimH, hlyA*	NR	
607	Dog	Not known	B2	13‐223	*cnf1, fimH, hlyA*	NR	
726	Cat	Chronic kidney disease	B2	43‐new	*cnf1, fimH, hlyA*	NR	
3710	Dog	Cystitis	B2	24‐new	*cnf1, fimH, hlyA*	NR	
3841, 3843	Cat	Acute kidney disease (3841), cystitis (3843)	B2	24‐13	*cnf1, pic, fimH, papC, hlyA, iucD*	NR	
3850	Cat	FLUTD	B2	103‐102	*cnf1, pic, fimH, hlyA*	NR	
14,057	Dog	Cystitis	B2	38‐9	*cnf1, fimH, hlyA*	NR	
8	Cat	Haematuria	B2	103‐21	*cnf1, fimH, hlyA*	NR	
3699	Dog	Haematuria	B2	24‐9	*cnf1, fimH, hlyA*	NR	
3068	Dog	Not known	B2	24‐10	*cnf1, pic, fimH, papC, hlyA, iucD*	NR	
4143	Cat	Cystitis	B2	24‐new	*cnf1, fimH, hlyA*	NR	
4154	Dog	Prostatitis	B2	103‐9	*cnf1, pic, fimH, hlyA*	NR	
473	Cat	Cystitis	B2	24‐21	*cnf1, fimH, hlyA*	NR	
2181	Dog	Haematuria	B2	13‐102	*astA, cnf1, pic, fimH, hlyA*	NR	
2484	Dog	Irregular bladder wall	B2	13‐27	*cnf1, fimH, hlyA*	NR	
2623	Dog	Dysuria	B2	4‐13	*astA, cnf1, pic, fimH, papC, hlyA, iucD*	NR	
2714	Dog	Haematuria	B1	103‐32	*cnf1, fimH, papC, hlyA, iucD*	AMP, TET, STX	*bla*_TEM,_ *bla*_ACT_ *, tet*(A), *dfrA5*
3248	Dog	Incontinence	B2	103‐9	*cnf1, fimH, hlyA*	AMP, TET, STX	*bla*_TEM_, *aad_A1_*, *tet*(A), *sul1, sul2*, *dfrA1, dfrA5*
3679	Dog	Not known	B2	24‐204	*cnf1, f17‐G, fimH, K88ab, hlyA*	AMP, CHL, STX	*bla*_TEM_, *sul2, cat*
703	Dog	Cystitis	B2	103‐new	*astA, cnf1, pic, fimH, hlyA*	NR	
1009	Dog	Dysuria	B2	13‐211	*astA, cnf1, fimH, hlyA*	NR	
494	Dog	Haematuria	B2	319‐306	*cnf1, fimH, hlyA*	NR	
1306	Dog	Bacteriuria	B2	24‐197	*cnf1, fimH, hlyA*	NR	
3000	Cat	Dysuria	B2	14‐197	*cnf1, fimH, hlyA*	NR	
4207	Cat	FLUTD	B2	14‐103	*astA, cnf1, pic, fimH, hlyA*	NR	
3383	Dog	Haematuria	B2	40‐30	*fimH, papC, hlyA, iucD*	AMP, CTX, CAZ, CIP, TET, GEN, FOF	*bla*_CTX‐M15_, *bla*_ACT_, *bla*_OXA‐2_, *tet*(A), *dfrA5, aac(3')‐IVa, aadA1*

Abbreviations: AMP, ampicillin; CTX, cefotaxime; CAZ, ceftazidime; CIP, ciprofloxacin; FLUTD, feline lower urinary tract disease; FOF, fosfomycin; GEN, gentamicin; NR, non‐resistant; SXT, trimethoprim/sulfamethoxazole; TET, tetracycline.

## 3 Results

45 out of 51 isolates were susceptible to all antibiotics tested. Among other isolates, resistance to ampicillin (*n* = 1), ampicillin, trimethoprim–sulfamethoxazole and tetracycline (*n* = 2), or ampicillin, trimethoprim–sulfamethoxazole and chloramphenicol (*n* = 1) was observed. One isolate displayed an ESBL phenotype and was resistant to ẞ‐lactams (exclusive of cefoxitin and carbapenems), ciprofloxacin (amino acid substitutions were detected in the QRDR of *gyrA* (83Ser→Leu and 87Asp→Asn) and *parC* (80Ser→Ile 84Glu→Val)), tetracycline, gentamicin, and fosfomycin. Four isolates were multidrug‐resistant (Sweeney et al. 2018) (Table 1). The detected resistance genes (*bla*_TEM_, *bla*_CTX_, *bla*_OXA‐2_, *sul1, sul2, dfrA1, dfrA5, tet*(A)*, aac(3')‐IVa, catA*) reflected the phenotypic findings well (Table 1). 50 out of 51 *E. coli* belonged to the phylogenetic group B2, and one isolate was assigned to phylogenetic group B1. Thirty‐four CH clonotypes were differentiated. CH103‐9 was the predominant clonotype (*n* = 7), along with more frequently occurring two isolates (CH24‐102 (*n* = 2); CH43‐1561(*n* = 2); CH13‐223 (*n* = 2); CH13‐5 (*n* = 2); CH13‐9 (*n* = 2); CH24‐13 (*n* = 2); CH38‐9 (*n* = 2)) showed clonal relatedness. An isolate belonging to B2‐CH40‐30 was positive for the O25b‐specific allele and confirmed as ST131 using seven loci MLST. All isolates showed the virulence factors *fimH* (51/51) and *hlyA* (51/51), determined via microarray. The virulence factors *cnf1* (50/51), *pic* (15/51), *papC* (11/51), and *iucD* (11/51) were also frequently detected, with co‐occurrence of *papC* and *iucD*.

## 4 Discussion

The present study aimed to characterise haemolytic *E. coli* isolated from samples of dogs and cats with urinary infections, using pheno‐ and genotyping methods. *E. coli* associated with UTI cases in Austrian cats and dogs are, until now, widely characterised (Loncaric et al. 2020; Marques et al. 2016).

In the present study, all but one isolate belonged to the phylogroup B2. *E. coli* isolates from the urinary tracts of humans and animals, particularly those causing ExPEC infections, are often linked to this phylogroup (Aurich et al. 2023; Siqueira et al. 2021; Jousserand et al. 2025; Valat et al. 2020; Osugui et al. 2014; Oh and Park, 2025; Johnson et al. 2009). In addition to *hlyA*, the adhesion gene *fimH*, which is referred to as a major virulence‐associated gene (VAG) of UPEC (Terlizzi et al. 2017), was observed in all isolates. Consistent with previous studies describing VAG's associated with UPEC from companion animals, the toxin‐encoding gene *cnf1* was observed in all but one isolate, while the *pic* gene (encoding a serine protease), the *iucD* gene (which is only found in virulent *E. coli* strains), and *papC* (encoding P‐fimbriae) were frequently detected (Aurich et al. 2023; Ksiezarek et al. 2021; Jousserand et al. 2025; Valat et al. 2020; Osugui et al. 2014; Johnson et al. 2009; Zogg et al. 2018). Although the predominance of phylogroup B2 was expected, the complete conservation of the *cnf1* and *hlyA* linkage within this geographical cohort offers novel insights into the regional clonal stability of UPEC. Other VAG's, were infrequently observed, such as *astA*, which encodes the enteroaggregative heat‐stable toxin EAST1, usually but not exclusively associated with Enteroaggregative *E. coli* (EAEC), or the K88 fimbriae (also known as F4 fimbriae) and f17‐G fimbriae. This is in concordance with other studies (Jousserand et al. 2025; Johnson et al. 2009). One isolate from the B1 phylogroup revealed associations with UPEC‐specific VAGs. While phylogroup B1 *E. coli* are typically considered commensal strains in the intestines of healthy humans and animals (Oh and Park 2025), they have been occasionally isolated from UTIs in companion animals (Aurich et al. 2023; Jousserand et al. 2025; Valat et al. 2020; Osugui et al. 2014; Oh and Park 2025; Johnson et al. 2009; Zogg et al. 2018).

Antibiotic phenotypic and genotypic resistance was a rare observation in the present study. While the majority of isolates (88.2%) were susceptible to all tested antibiotics, four out of six isolates displayed a multidrug‐resistant profile (Sweeney et al. 2018). This low antibiotic resistance rate in canine and feline UPEC isolates, mainly belonging to the phylogroup B2, is consistent with the resistance situation observed in previous studies (Aurich et al. 2023; Jousserand et al. 2025; Valat et al. 2020; Zogg et al. 2018). The detection of a single isolate (3383, displaying an ESBL phenotype) belonging to B2 (phylotype) ‐O25b, (serotype) ‐ST131, (sequence type), and ‐CH40‐30 (clonotype), carrying *bla*_CTX‐M‐15_ and *bla*_OXA‐2_, and exhibiting ciprofloxacin resistance, represents an isolated observation that warrants attention. Recognised as a major human‐associated “high‐risk” pandemic pathogen, the B2‐O25b‐ST131 clone has been universally classified as an ExPEC and/or UPEC and is responsible for various infections in humans and animals, including those with UTIs (Jousserand et al. 2025; Clark and Maresso 2021; Rogers et al. 2011; Nicolas‐Chanoine et al. 2014; Bonnet et al. 2021; Pitout and Finn 2020). Until present, among animals in Austria, the B2‐O25b‐ST131 clone has been sporadically detected from Austrian animals, including wildlife (Loncaric et al. 2013), canine prostate (Loncaric et al. 2020), porcine intestine–associated samples (Bernreiter‐Hofer et al. 2021), and very recently from a faecal sample (Saria et al. 2025). Besides B2‐O25b‐ST131‐CH40‐30, belonging to *E. coli* clonal complex (CC) 131, other clonotypes detected in the present study have also been observed in UPECs from recent studies. These are CH13‐27 and CH13‐223 belonging to *E. coli* CC12, CH24‐9, CH24‐10, CH24‐13, CH24‐102, and CH24‐1431 to CC73; CH14‐2 to CC127; CH13‐5 to CC141; and, finally, CH103‐9 to CC372 (Jousserand et al. 2025; Valat et al. 2020; Oh and Park 2025; Zogg et al. 2018). While *E. coli* isolates belonging to CC131, CC73, CC12, and CC141 have been reported as commonly isolated from humans (Manges et al. 2019; Emery et al. 2023; Riley 2014), isolates belonging to CC372 have been primarily associated with dogs (Aurich et al. 2023; Jousserand et al. 2025; Valat et al. 2020).

This study has several limitations. First, our analysis has inherent selection bias due to restriction to haemolytic *E. coli*; consequently, the virulence and resistance potential of non‐haemolytic strains causing UTIs require further investigation. Second, the panel of selected VAGs was limited. These data could be enriched in a future study by including a larger collection of strains and utilising whole‐genome sequencing for a more comprehensive characterisation. Finally, the low presence of resistant isolates restricts broader epidemiological insights into antimicrobial resistance within canine and feline UTIs.

## 5 Conclusions

Although the majority of isolates were susceptible to all tested antibiotics, the virulence potential of these strains poses a relevant concern for both public and animal health. Consequently, the risk of human infection or colonisation from these isolates remains to be fully determined. In a clinical context, our data indicate that a significant proportion of *E. coli* UTIs in companion animals can still be successfully managed with existing antibiotic therapies. Nevertheless, the existence of multi‐drug‐resistant pet *E. coli* strains with a zoonotic potential underscores the critical need for a continuous “One Health” approach to monitor and control their virulence and antimicrobial resistance.

## Author Contributions

**Sarah Büttner**: Formal Analysis, Investigation, Visualization, Writing – Original Draft Preparation. **Joachim Spergser**: Funding Acquisition, Resources, Methodology, Writing – Review & Editing. **Olga Makarova**: Data Curation, Methodology, Validation, Writing – Original Draft Preparation, Writing – Review & Editing. **Michael P. Szostak**: Data Curation, Investigation, Validation, Writing – Review & Editing. **Adriana Cabal Rosel**: Investigation, Methodology, Writing – Review & Editing. **Werner Ruppitsch**: Investigation, Methodology, Writing – Review & Editing. **Sabine Schäfer‐Somi**: Resources, Validation, Writing – Review & Editing. **Elke Müller**: Formal Analysis, Investigation, **Sascha D. Braun**: Investigation, Methodology, Writing – Review & Editing. **Stefan Monecke**: Conceptualization, Methodology, Writing – Review & Editing. **Ralf Ehricht**: Conceptualization, Resources, Methodology, Writing – Review & Editing. **Frank Künzel**: Conceptualization, Investigation, Resources, Validation, Writing – Review & Editing. **Igor Loncaric**: Conceptualization, Formal Analysis, Investigation, Methodology, Supervision, Validation, Visualization, Writing – Original Draft Preparation, Writing – Review & Editing.

## Funding

The authors have nothing to report.

## Ethics Statement

All samples were received from third parties and, therefore, are not subject to the reporting obligations of the Ethics and Animal Welfare Commission of the University of Veterinary Medicine in Vienna.

## Supporting information




**Table S1**: All clinical data

**Table S2**: Virulence‐associated genes tested
